# Radiolucency around highly porous sockets and hydroxyapatite-coated porous sockets in total hip arthroplasty for hip dysplasia

**DOI:** 10.1007/s00590-018-2351-3

**Published:** 2018-11-28

**Authors:** Hiroshi Imai, Joji Miyawaki, Tomomi Kamada, Akira Maruishi, Jun Takeba, Hiromasa Miura

**Affiliations:** Department of Bone and Joint Surgery, Ehime University Graduate School of Medicine, Shitsukawa, Toon, Ehime 791-0295 Japan

**Keywords:** Cementless total hip arthroplasty, Developmental dysplasia of the hip, Highly porous socket, Hydroxyapatite-coated porous socket, Radiolucent line, Osseointegration

## Abstract

Studies over the past decade have reported that the use of highly porous sockets in total hip arthroplasty (THA) results in osseointegration and long-term implant stability. However, some reports have raised concerns regarding radiographic evidence of poor osseointegration with features of fibrous tissue ingrowth. The purpose of this study was to compare clinical and radiographic assessments of highly porous sockets with those of hydroxyapatite (HA)-coated porous sockets in THA for hip dysplasia (DDH) at least 1 year after surgery. A total of 127 patients (136 hips) were recruited for the study. Of these, 94 patients (101 hips) received highly porous sockets with clustered screws, while 33 patients (35 hips) received HA-coated porous sockets with clustered screws. There was no difference in clinical outcomes between the two types of sockets. All HA-coated porous sockets were radiographically stable, without radiolucent lines. Fifteen hips had radiolucent lines in two or three DeLee and Charnley zones, accompanied by sclerotic lines along the circumferences of the highly porous sockets. A significant difference in the height of the preoperative osteophyte of the anterior acetabular wall was observed between 86 hips with one or no radiolucent lines and 15 hips with two or three radiolucent lines. In cases of DDH with atrophic bone remodeling pattern, highly porous sockets with multiple screws may be used, while HA-coated porous sockets with clustered screws result in better sealing of the bone–component interface.

## Introduction

Failed total hip arthroplasties (THAs) pose greater clinical and economic burdens than do failed total knee arthroplasties [1]. Aseptic loosening of the acetabular component is one of the most common causes of THA failure [2]. Polyethylene wear leading to periacetabular osteolysis and failed initial stability of the acetabular component are major sources of concern in primary THA, and are caused by aseptic loosening of cementless acetabular components. It was recently shown that highly cross-linked polyethylene may be associated with reduced wear, osteolysis, and revision rates compared with conventional polyethylene in THA patients. Hence, the initial stability of the acetabular component is the most critical factor for osseointegration in primary and revision THA. This stability is markedly influenced by the coefficient of friction of the acetabular component in THA [3, 4]. Micromotion greater than 150 μm at the interface between host bone and implant has been shown to create a fibrous tissue ingrowth pattern and the potential for implant instability [5]. An unstable initial fit is one factor that may result in unsuccessful implantation.

Acetabular components using metal implants with highly porous surfaces were developed not only to increase surface area for tissue ingrowth but also to increase the surface roughness of the component [6]. Increasing the coefficient of friction can potentially eliminate micromotion at the interface. Highly porous metal acetabular sockets have been introduced and have reportedly led to osseointegration in primary and revision hip arthroplasty as well as immediate and long-term implant stability [7]. The highly porous metals presently used are manufactured from tantalum or titanium. Trabecular titanium, which has a solid cellular structure that is highly porous, is designed with multi-planar hexagonal interconnected cells to mimic the trabecular morphology of natural bone [8]. These materials result in a near-physiological modulus of elasticity (1.5–3 GPa) [9], and good results are achievable even when less than 50% of host bone remains [10]. The use of highly porous tantalum sockets for developmental dysplasia of the hip (DDH) provided satisfactory 10-year clinical and radiographic results, though it is still challenging to perform successful cementless socket fixation during THA for DDH [11]. Importantly, one study reported that the coefficient of friction provided by highly porous metal acetabular components (DePuy Pinnacle Gription) did not provide better resistance to migration under a bending load when compared with a standard porous coated component [12]. Another study demonstrated concerning radiographic evidence of poor osseointegration with features of fibrous tissue ingrowth [13].

The purpose of this study was to compare the clinical and radiographic evaluations of highly porous titanium sockets with hydroxyapatite (HA)-coated porous sockets in cementless THA for DDH at least 1 year after the primary operation.

## Patients and methods

We performed a retrospective study comparing the intermediate-term with short-term clinical and radiographic outcomes of DDH treated with THA using either highly porous sockets or HA-coated porous sockets.

This study was approved by our institution’s scientific research board, and conducted in accordance with the World Medical Association Declaration of Helsinki Standard of 1964, as revised in 1983 and 2000. All patients were informed about the study in detail before providing written informed consent for enrollment, including consent for postoperative computed tomography (CT) imaging.

From December 2014 to December 2016, we used a highly porous socket and a shorter, tapered, broach-only femoral stem, while from January 2017 to May 2017 we used an HA-coated porous socket and the same femoral stem. Of the 156 consecutive patients (165 hips) who underwent cementless THA and could be observed for 1 year or more, 127 patients (136 hips) were recruited for this study. The following cases were excluded: seven patients (seven hips) who did not attend all follow-up appointments, two patients (two hips) who died for any cause, 12 patients (12 hips) with osteonecrosis of the femoral head, two patients (two hips) with rheumatoid arthritis, two patients (two hips) with posttraumatic osteoarthritis, one patient (one hip) with septic arthritis, and three patients (three hips) who received the other socket and a changeable neck stem due to a stem anteversion angle greater than 55° as seen during preoperative three-dimensional (3D) computerized planning.

The mean age of the subjects at the time of surgery was 61.0 years (standard error [SE] 1.2 years, range 40–86 years). There were 21 male patients (21 hips) and 106 female patients (115 hips). The mean duration of follow-up after surgery was 1.6 years (SE 0.06, range 1.1–3.5 years). DDH was classified using the Crowe et al. classification [14]. Based on this classification, 115 hips in our group had Group 1 DDH, 17 had Group 2 DDH, four had Group 3 DDH, and there were no cases of Group 4 DDH. The canal flare index (CFI) was determined using the Noble et al. classification [15]. The cortical index (CI) was defined using the Dorr et al. classification [16]. We aimed for a socket center–edge angle of greater than 5° [17]. Finally, 100 patients (104 hips) with dysplastic acetabula were managed with autologous morselized bone grafts in the gap between the host bone and the lateral margin of the socket. No patients received autologous block bone grafts.

**Fig. 1:**
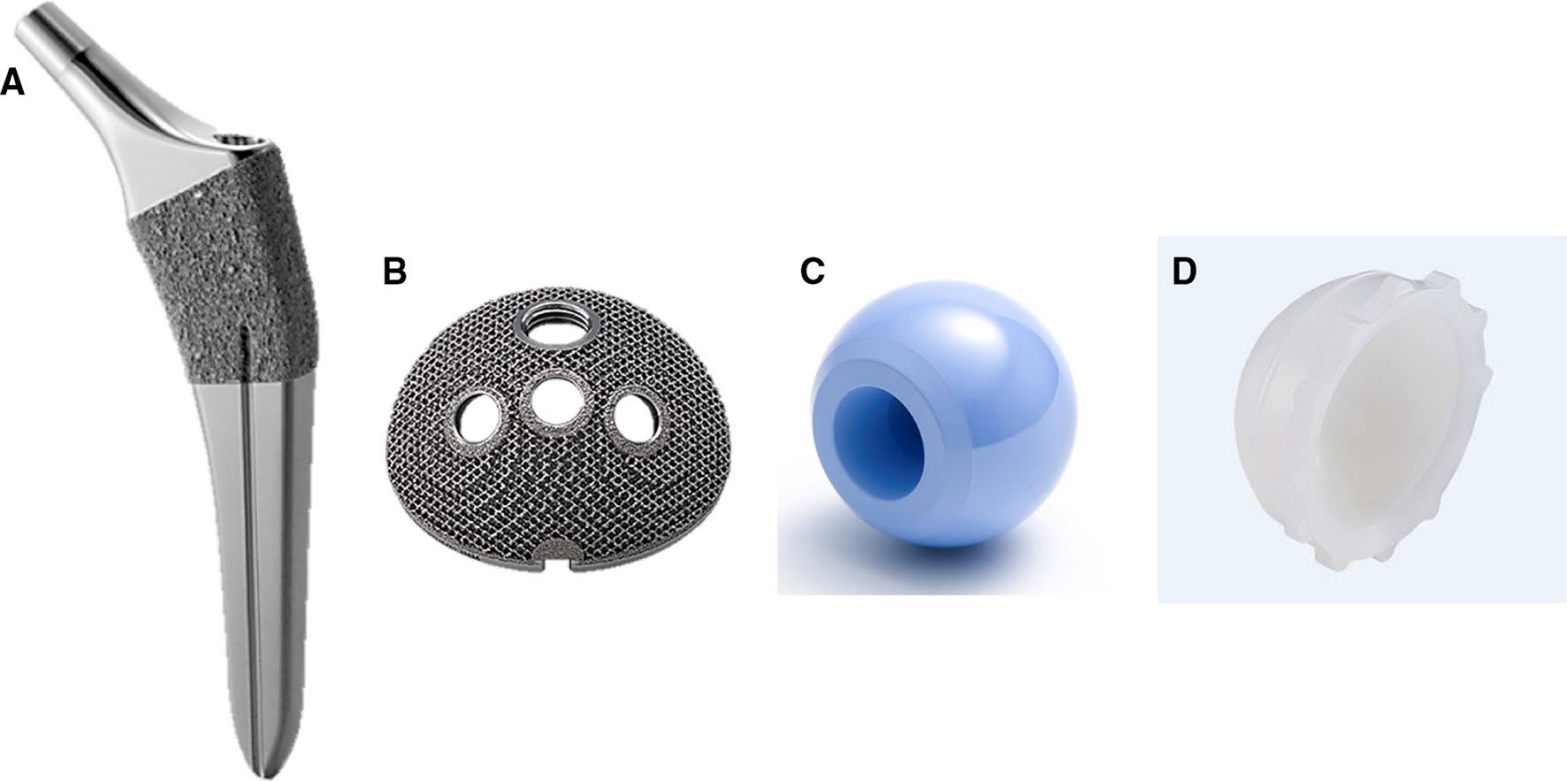
**a** A hydroxyapatite-coated, shorter, tapered wedge stem (J-Taper™ HA stem; Kyocera Medical, Osaka, Japan). **b** A porous, hexagonal, tridimensional, multiplane structure obtained using an electron beam melting technique (SQRUM TT™ socket; Kyocera Medical). **c** A 28/32-mm, zirconium-toughened, aluminum femoral head (Bioceram^®^ AZUL; Kyocera Medical). **d** A cross-linked polyethylene liner grafted with biocompatible phospholipid polymer (AQUALA^®^; Kyocera Medical)

We used two combinations of components, with acetabular sockets that had the same geometry and differed only in the coating used. One combination, used in 94 patients (101 hips), was made of titanium alloy (Ti6Al4V) and consisted of a porous hexagonal tridimensional multiplane structure obtained using an electron beam melting technique (SQRUM TT™ socket; Kyocera, Kyoto, Japan), a 28/32-mm zirconium-toughened aluminum femoral head (Bioceram^®^ AZUL; Kyocera, Kyoto Japan), a cross-linked polyethylene liner grafted with biocompatible phospholipid polymer [18] (AQUALA^®^; Kyocera), and an HA-coated shorter, tapered wedge stem (J-Taper™ HA stem; Kyocera), as shown in Fig. 1a–d. The SQRUM TT™ socket has a hemispherical socket, 60% porosity, a coefficient of friction of 1.09, and an average pore size of 640 microns, and allows for supplementary fixation by 6.5-mm cancellous bone screws. The component is available as a hemispherical no-hole socket, a cluster-hole socket (with clustered screw holes), and hemispherical multiple-hole socket (with multiple screw holes). In this study, we used the cluster-hole socket with three screws in all cases.

**Fig. 2:**
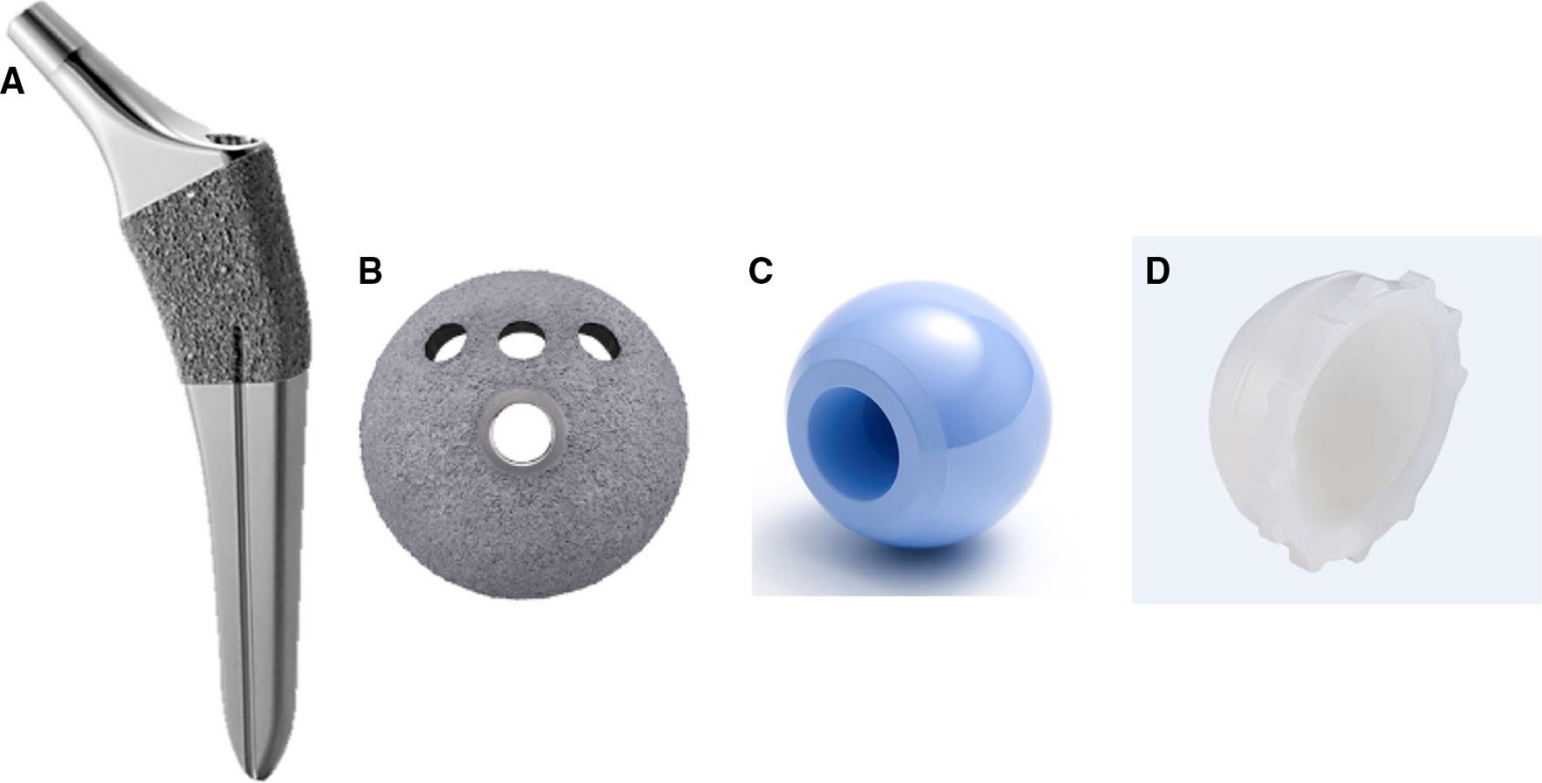
**a** A hydroxyapatite-coated, shorter, tapered wedge stem (J-Taper™ HA stem; Kyocera Medical, Osaka, Japan). **b** A hydroxyapatite-coated porous socket (SQRUM HA™ socket; Kyocera Medical). **c** A 28/32-mm, zirconium-toughened, aluminum femoral head (Bioceram^®^ AZUL; Kyocera Medical). **d** A cross-linked polyethylene liner grafted with Biocompatible Phospholipid Polymer (AQUALA^®^; Kyocera Medical)

The second combination, used in 33 patients (35 hips), was made of titanium alloy (Ti6Al4V) and comprised an HA-coated porous socket with clustered screw holes (SQRUM HA™ socket; Kyocera) and the same femoral head, a polyethylene liner, and a tapered wedge stem as shown in Fig. 2a–d. The SQRUM HA™ socket has a hemispherical socket, 50% porosity, a coefficient of friction of 0.56, and an average pore size of 380 microns, and allows for supplementary fixation by 6.5-mm cancellous bone screws. The component is available as a hemispherical no-hole socket, a cluster-hole socket, and a hemispherical multiple-hole socket. In this study, we used the cluster-hole socket with three screws in all cases. The demographic data of the two groups of patients are summarized in Table 1.

**Table 1:** Patient demographic data

	Highly porous socket (101 hips)	Hydroxyapatite-coated porous socket (35 hips)	*p* values
Age (years)^c^	62.5 ± 1.1 (38–86)	64.5 ± 1.9 (40–86)	N.S.^a^
BMI (kg/m^2^)^c^	25.4 ± 0.5 (17.0–39.0)	24.7 ± 0.6 (18.0–33.0)	N.S.^a^
Crowe G1/2/3/4	84/13/4/0 (101 hips)	31/4/0/0 (35 hips)	N.S.^b^
Cortical index^c^	0.52 ± 0.008 (0.20–0.68)	0.54 ± 0.01 (0.39–0.64)	N.S.^a^
Canal flare index^c^	3.6 ± 0.07 (1.8–5.6)	3.6 ± 0.07 (2.8–4.6)	N.S.^a^

^a^Unpaired *t* test

^b^Chi-square test

^c^Values are expressed as the mean ± standard error, with range in parentheses

The surgery was performed by one senior surgeon. All operations were performed while the patient was in the lateral position using a modified Watson-Jones approach [19]. After the initial skin incision, made according to the anatomical configuration of the femur, the stem was fixed using the press-fit method in order to achieve a strong initial fixation to the bone. We then measured the anteversion angle of the stem and prepared the socket. For socket placement, we used a CT-based fluoroscopy matching navigation system (VectorVision Hip 3.5.1; Brainlab, Feldkirchen, Germany). A ball used for the infrared light navigation system was fixed with two pins onto the iliac crests. The acetabulum was under-reamed by 1 mm in both the SQRUM TT™ and the SQRUM HA™ socket. Next, a socket of the planned size was fixed using the press-fit method. The insertion angles were set to 40° for radiographic inclinations and to the planned angles for radiographic anteversions, which were adjusted based on the angle of anteversion of the stem that was determined based on combined anteversion theory [20–24]. In 78 patients (81 SQRUM TT™ sockets) and 22 patients (23 SQRUM HA™ sockets), the initial fixation was achieved using three screws and placement of morselized autologous bone grafts against the superolateral part of the ilium above the acetabular socket. After implant placement, a drainage tube was placed and the wound was closed. On postoperative day 1, if patients were in good overall clinical condition, the drain was removed and gait training with full weight bearing was initiated.

Clinical assessment was completed twice by two orthopedic surgeons, each of whom had more than 15 years of experience in assessing hip function. The time between measurements was at least 2 weeks. Both surgeons were blinded to the radiographic results at the time of the evaluation. They used the Japanese Orthopaedic Association’s scoring system to evaluate hip joint function [25] and investigated the incidence of postoperative complications. The JOA hip evaluation system consists of a 100-point scale comprising the following subcategories: pain (0–40 points), ability to walk (0–20 points), range of motion (0–20 points), and ability to complete daily living tasks (0–20 points). Higher scores indicated better conditions. Scores at the final follow-up were compared to the preoperative scores.

We assessed the fixation of the socket and the stem. Radiolucent lines and osteolytic lesions in the three acetabular zones of DeLee and Charnley were recorded [26], and enlargement of the radiolucent lines around the socket was evaluated using the five grades of Long and Dorr [27]. Socket migration was defined as a change in the position of the acetabular component of more than 2 mm or a change in socket inclination of more than 5° [28]. The femoral stem was evaluated with regard to the presence of radiolucent lines, osteolysis, cancellous condensation, cortical hypertrophy, reactive lines, and pedestal formations according to the criteria defined by Engh et al. [29]. All the radiographic and CT measurements were taken twice by both orthopedic surgeons, each of whom had more than 15 years of experience in assessing radiographic and CT evaluation. The time between measurements was at least 2 weeks. Both surgeons were blinded to the clinical results at the time of the evaluation.

Three-dimensional CT scans were performed using a Philips Brilliance 64 scanner (Marconi Medical System, Best, Netherlands). The scanning technique parameters were: 120 kV, 150–250 effective mAs (depending on the patient’s size), and 0.5 s rotation time. Contiguous slices (2.0 mm) were obtained from the bilateral anterior superior iliac spines to the distal end of the femur, with the patient in a supine position with the hips extended and thighs horizontal and parallel. All raw CT scan data, which are in the Digital Imaging and Communications in Medicine (DICOM) format, were entered into an available planning software for the Kyocera 3D-template^®^.

### Statistical analysis

The normality of continuous data was assessed with Levene’s test. Since the data were normally distributed, the unpaired Student’s *t* test was used. Intraobserver variances for the JOA hip score were determined by comparing separate radiographic and CT assessments of the same patient by the same observer with at least a 2-week interval between assessments. Intraobserver and interobserver variances in the JOA hip score were determined by comparing radiographic and CT measurements and expressed using interclass correlation coefficients (ICC), with ICC < 0.20 indicating slight agreement, 0.21–0.40 fair agreement, 0.41–0.60 moderate agreement, 0.61–0.80 substantial agreement, and > 0.80 almost perfect agreement [30]. SPSS for Windows version 20 (IBM, Armonk, NY) was used for all statistical analyses. A *p* value of < 0.05 was used to indicate statistical significance.

## Results

The JOA hip scores improved significantly from 52.2 points (SE 1.5, range 24–79) preoperatively to 88.7 points (SE 1.2, range 55–99) postoperatively in patients with the SQRUM TT™ socket. The scores improved significantly from 54.2 (SE 1.8, range 40–77) preoperatively to 87.0 points (SE 1.6, range 70–100) postoperatively in patients with the SQRUM HA™ socket. No differences between the highly porous sockets and the HA-coated porous sockets were observed in terms of clinical outcomes.

Two intraobserver ICCs were calculated; both were 0.98. The interobserver ICC was 0.86. These values indicate almost perfect agreement in JOA hip score measurements.

None of the patients developed postoperative infections, paralysis, deep vein thrombosis, or dislocation.

**Fig. 3:**
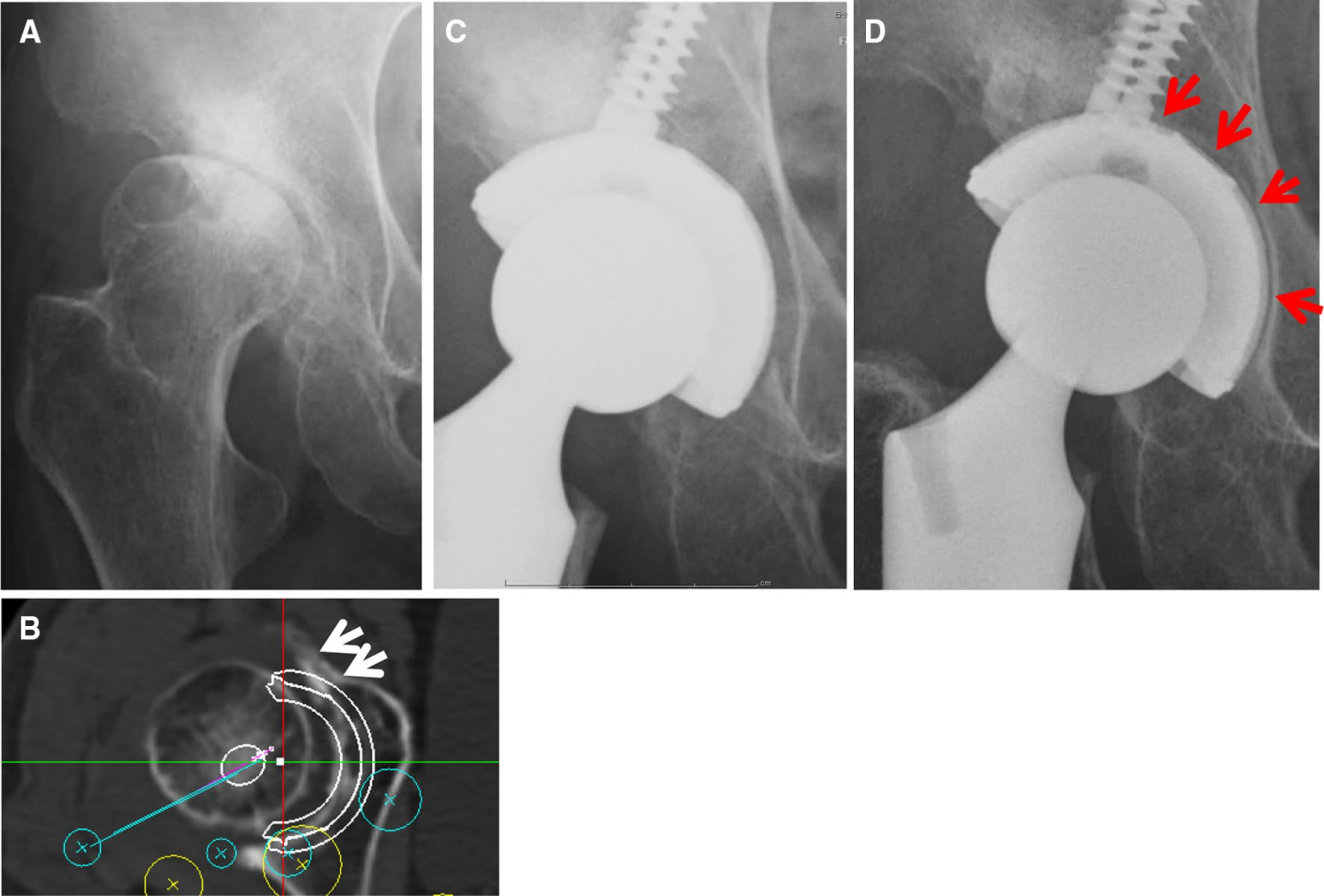
**a** A radiograph image of a 62-year-old-female patient with developmental dysplasia of the hip with atrophic bone remodeling pattern. Preoperative anteroposterior view. **b** The preoperative osteophyte of the anterior acetabular wall (white arrow). Preoperative CT image. **c** Postoperative anteroposterior view at 1-week follow-up. THA was used a highly porous socket with clustered screws (SQRUM TT™ socket; Kyocera Medical). There were no initial gaps between the acetabular socket and the acetabular host bed. **d** At 2-year follow-up, the radiolucent lines were seen in two DeLee and Charnley zones (red arrow)

In the radiological evaluations, there were no initial gaps between the outer surface of the two types of acetabular sockets and the acetabular host bed immediately postoperatively. But at 1 year after surgery, 32 hips demonstrated radiolucent lines accompanied by sclerotic lines on the circumferences of the SQRUM TT™ sockets with clustered screws. Radiolucent lines were seen in only one DeLee and Charnley zone in 17 hips, in two zones in 13 hips, and in three zones in two hips, as shown in Fig. 3a–d. At 2 years after surgery, there was no enlargement of the radiolucent lines accompanied by sclerotic lines on the circumferences of the SQRUM TT™ sockets with clustered screws. On the other hand, patients with SQRUM HA™ sockets with clustered screws demonstrated no initial gaps immediately postoperatively, no radiolucent lines and no periacetabular osteolysis in any of the three DeLee and Charnley zones for the entire follow-up period. No socket migration was observed in any hips (Table 2). All autologous morselized bone grafts in the sockets incorporated without collapse and without resorption.

**Table 2:** Modified DeLee/Charnley skeletal fixation score for acetabulum

	Fixation grade	Radiolucency by zone	Highly porous cup (101 hips)	HA-coated cup (35 hips)
Bone ingrowth, stable	IA	None	69	35
	IB	One zone	17	0
	IC	Two zones	13	0
Fibrous ingrowth, stable	II	Complete RLL < 2 mm all zones	2	0
Fibrous fixation, unstable	III	Progressive RLL zone 3, complete RLL ≥ 2 mm all zones, or socket migration	0	0

We compared the 86 hips with one or no radiolucent lines and the 15 hips with two or three radiolucent lines. There was a significant difference between the groups in terms of the height of the preoperative osteophyte of the anterior acetabular wall (*p* < 0.05), but no significant difference in age, body mass index, Dorr classification, Noble classification, socket CE angle, or bone graft in the gap between the host bone and the lateral margin of the socket (*p* = 0.63, *p* = 0.17, *p* = 0.84, *p* = 0.93, *p* = 0.06, and *p* = 0.17, respectively) (Table 3). No radiolucent lines or osteolysis at the bone–stem interface and no subsidence or loosening were evident on any of the radiographs.

**Table 3:** Relationships between 86 hips with one or no radiolucent lines and 15 hips with two or three radiolucent lines

	Fixation grade (1a and 1b)	Fixation grade (1c and 2)	*p*
Age (years)^c^	62.3 ± 1.2 (38–85)	63.4 ± 2.4 (54–86)	N.S.^a^
BMI (kg/m^2^)^c^	25.6 ± 0.5 (17.0–39.0)	23.8 ± 1.3 (17.0–34.0)	N.S.^a^
Follow-up period (months)	25.7 ± 0.8 (14–40)	26.3 ± 2.0 (17–40)	N.S.^a^
Cortical index^c^	52.3 ± 0.9 (20.0–68.0)	51.8 ± 1.5 (41.0–58.4)	N.S.^a^
Canal flare index^c^	3.6 ± 0.1 (2.0–6.0)	3.6 ± 0.2 (2.5–4.5)	N.S.^a^
Socket CE angle (°)^c^	26.9 ± 1.1 (5.3–48.3)	24.3 ± 2.9 (17.0–31.0)	N.S.^a^
Bone graft	67/86	14/15	N.S.^b^
Height of osteophyte (mm)^c^	14.3 ± 1.2 (0–36)	8.1 ± 2.6 (0–29)	< 0.05^a^

^a^Unpaired *t* test

^b^Chi-square test

^c^Values are expressed as the mean ± standard error, with range in parentheses

The Kyocera 3D-template^®^ analysis showed that the postoperative insertion angles of the sockets were as follows: for the SQRUM TT™ socket, the radiographic inclination was 41.9° (SE 0.4°, range 33–48.5°) and the radiographic anteversion was 13.3° (SE 0.8°, range − 2.7° to 31.8°), while for the SQRUM HA™ socket the radiographic inclination was 41.9° (SE 0.8°, range 35.0–51.5°) and the radiographic anteversion was 16.9° (SE 1.4°, range − 10.0° to 22.5°). No significant difference was observed between the highly porous socket and the HA-coated porous socket in either the radiographic inclination or the radiographic anteversion angle.

Two intraobserver ICCs were calculated; both were 0.9 or more for the radiographic and CT measurements. The interobserver ICCs were also 0.8 or more. These values indicate almost perfect agreement between different radiographic and CT measurements.

## Discussion

In THA, highly porous metal acetabular sockets are designed to enhance osseointegration, reduce stress shielding with a modulus of elasticity closer to bone [31], and increase stability of the initial interference fit with a higher friction coefficient [32].

The highly porous metals presently used are manufactured from tantalum or titanium. Compared with conventional cementless porous designs, they have a higher coefficient of friction and modulus of elasticity and greater porosity (60–80%). The porosity of tantalum is approximately two to three times greater than that of cobalt chromium and titanium mesh; this results in greater bone and fibrous tissue ingrowth potential, which is extremely valuable if there is limited host bone [11, 33–35]. The modulus of elasticity of porous tantalum is similar to that of subchondral bone, thus allowing for a physiological transfer of load from implant to host bone, minimization of stress shielding, and preservation of bone stock [36]. The SQRUM TT™ socket is a 3D metal interface that has also been used for porous biological fixation. It is similar in structure to porous tantalum acetabular sockets.

In this study, 32 hips had both radiolucent and sclerotic lines on the circumferences of the SQRUM TT™ sockets with clustered screws; these lines occurred in two zones in 13 hips and in three zones in two hips. During the entire follow-up period, the SQRUM HA™ sockets with clustered screws exhibited no radiolucent lines and no periacetabular osteolysis in any of the three DeLee and Charnley zones. All HA-coated cups remained radiographically stable without radiolucent lines. But the number of the SQRUM HA™ sockets in THA for DDH (*n* = 35) was fewer than the SQRUM TT™ sockets (*n* = 101); therefore, a consequence adverse to this study may be induced in the future.

The SQRUM TT™ socket with clustered screws was developed not only to increase the surface area for tissue ingrowth, but also to increase the surface roughness of the component [6, 22, 23]. However, in some cases this socket did not result in good osseointegration in dysplastic hips, with the low height of the preoperative osteophyte of the anterior acetabular wall. It is possible that the SQRUM TT™ socket with clustered screws caused micromotion between the socket and the bone in DDH with atrophic bone remodeling pattern around the socket in the early stage after surgery. When the SQRUM TT™ socket with clustered screws fails to achieve initial stability in THA for DDH, poor osseointegration with features of fibrous ingrowth may occur, observable radiographically, resulting in future loosening and migration of the socket. In cases of DDH where the host bone exhibits an atrophic remodeling pattern, we should consider using a SQRUM TT™ socket with multiple screws.

In contrast to the above findings, all SQRUM HA™ sockets with clustered screws resulted in radiographic stability without radiolucent lines. Previous studies reported that HA improved results by facilitating more complete ingrowth of bone and better sealing of the interface at 2- and 5-year follow-up [37–39]. Therefore, in cases of DDH where there is atrophic bone remodeling pattern, it would be optimal to use highly porous sockets with multiple screws or HA-coated porous sockets with clustered screws, since both have an advantage in terms of better sealing of the bone–component interface.

This study had several limitations. First, 17 patients (17 hips) were excluded due to concurrent conditions, as detailed above. Second, our results were not fully conclusive due to the small number of cases (*n* = 136). Third, the number of the SQRUM HA™ sockets in THA for DDH (*n* = 35) was fewer than that of the SQRUM TT™ sockets (*n* = 101) in spite of comparative study. Fourth, the short-term follow-up period made it difficult to compare differences in functional outcomes. Finally, the retrospective design of the study yielded an evidence grade of only 3.

## Conclusion

We found that in patients with DDH who underwent THA, HA-coated porous sockets with clustered screws were better than highly porous sockets with clustered screws in terms of the intermediate-term with short-term radiographic results. The circumferences of highly porous sockets demonstrated radiolucent and sclerotic lines in 32 hips; these lines occurred in two zones in 13 hips and in three zones in two hips. It is possible that the highly porous sockets with clustered screws caused micromotion between the socket and the bone in DDH with the low height of the preoperative osteophyte of the anterior acetabular wall in the early stage after surgery. Therefore, in DDH with atrophic bone remodeling pattern, highly porous sockets with multiple screws or HA-coated porous sockets with clustered screws should be used since these have the advantage of better sealing the bone–component interface.
